# Methicillin-Resistant *Staphylococcus pseudintermedius* in Rats

**DOI:** 10.3201/eid1901.120897

**Published:** 2013-01

**Authors:** Chelsea Gardner Himsworth, David M. Patrick, Kirbee Parsons, Alice Feng, J. Scott Weese

**Affiliations:** Author affiliations: University of British Columbia, Vancouver, British Columbia, Canada (C.G. Himsworth, D.M. Patrick, K. Parsons, A. Feng);; British Columbia Ministry of Agriculture, Abbotsford, British Columbia, Canada (C.G.Himsworth);; University of Guelph, Guelph, Ontario, Canada (J.S. Weese)

**Keywords:** Methicillin resistance, Staphylococcus pseudintermedius, rats, Norway rats, antimicrobial resistance, bacteria, staphylococci, Rattus spp., Rattus norvegicus

**To the Editor**: *Staphylococcus pseudintermedius* is a coagulase-positive species in the *S. intermedius* group. Previously misidentified as *S. intermedius*, *S. pseudintermedius* is now recognized as a leading cause of opportunistic infection in dogs (*1*) and a cause of sporadic infections in other species, including humans (*1*,*2*). Additionally, evidence of zoonotic transmission of *S.* *pseudintermedius* from dogs to humans has been reported (*3*,*4*). Although information regarding the pathogenic process of *S. pseudintermedius* is limited, the bacterium is known to possess virulence factors similar to those found in *S. aureus*, including a leukotoxin comparable to the Panton-Valentine leukocidase associated with community-acquired *S. aureus* infection (*1*).

Of concern is the emergence and widespread international recognition of methicillin-resistant *S. pseudintermedius* (MRSP) (*1*). One veterinary laboratory noted a 272% increase in MRSP cases from 2007–2008 through 2010–2011 (*5*). As with methicillin-resistant *S. aureus*, MRSP resistance is conferred by the *mecA* gene, making MRSP resistant to all β-lactam antimicrobial drugs and some other antimicrobial drug classes (*1*). Compared with methicillin-susceptible strains, MRSP seems better able to colonize humans (*3*).

The potential for zoonotic transmission and concerns that MRSP could be mistaken for other methicillin-resistant staphylococci (*1*,*2*) suggest the need for further investigation into the epidemiology of this pathogen. One question yet to be addressed is whether commensal pests, particularly rats (*Rattus* spp.), could serve as a source of MRSP because of their pervasiveness, their propensity toward close contact with humans, and the fact that they are the source of several other zoonotic diseases (*6*). We report MRSP carriage in wild Norway rats (*R. norvegicus*) in Vancouver, British Columbia, Canada.

During September–November 2011, Norway rats were trapped in a random sample of alleys in Vancouver’s Downtown Eastside, an impoverished neighborhood with high levels of homelessness, intravenous drug use, and HIV infection. Immediately after the rats were euthanized, a sterile swab was used to sample the oropharynx and nares of each rat. 

Swabs were placed in 2 mL of enrichment broth containing 10 g/L tryptone T, 75 g/L sodium chloride, 10 g/L mannitol, and 2.5 g/L yeast extract and incubated for 24 h at 35°C. Aliquots of 100 µL were streaked onto mannitol salt agar with 2 µg/mL oxacillin and incubated at 35°C for 48 h. Suspected staphylococcal isolates were subcultured onto Columbia blood agar and identified according to colony morphologic appearance, Gram staining, and catalase reaction. Tube coagulase-positive isolates were speciated by using a multiplex PCR specific for the thermonuclease (*nuc*) gene (*7*). Methicillin resistance was confirmed by demonstrating penicillin-binding protein 2a antigen with the latex-agglutination test (Oxoid Ltd., Basingstoke, UK). Isolates were typed by sequencing of the *mec-*associated direct repeat unit (*dru* typing) (*8*). Antimicrobial drug susceptibility was evaluated by broth microdilution (Sensititre; Trek Diagnostics, Cleveland, OH, USA), according to Clinical and Laboratory Standards Institute guidelines (www.clsi.org/). The study was approved by the University of British Columbia Animal Care Committee.

MRSP was isolated from 5 (2.1%) of 237 rats trapped. However, lack of standardized screening methods for MRSP could have resulted in underestimation of MRSP prevalence. Of the 5 isolates, 3 were *dru* type dt11a, a strain commonly found in dogs (*8*), and the other 2 were a novel *dru* type (assigned dt7ac). All isolates tested demonstrated resistance to multiple antimicrobial drug classes (Table).

**Table:** Sensitivity profiles for methicillin-resistant *Staphylococcus pseudintermedius* isolated from wild Norway rats, Vancouver, British Columbia, Canada*

Drug	Isolate no. (strain type)†			
	1 (dt11a)	2 (dt11a)	3 (dt11a)	4 (dt7ac)
β-lactams	R	R	R	R
Clindamycin	R	R	R	R
Erythromycin	R	R	R	R
Daptomycin	S	S	S	S
Linezolid	S	S	S	S
Gentamicin	R	S	S	S
Tetracycline	S	S	S	R
Tigecycline	S	S	S	S
Trimethoprim–sulfamethoxazole	S	S	S	S
Quinupristin–dalfopristin	R	S	S	S
Rifampin	R	S	S	S
Chloramphenicol	S	S	S	S
Nitrofurantoin	S	S	S	S
Ciprofloxacin	R	S	S	S
Levofloxacin	I	S	S	S
Moxifloxacin	I	S	S	S
Vancomycin	S	S	S	S

*Of 5 isolates, 1 was not available for antimicrobial susceptibility testing. R, resistant; S, sensitive; I, intermediate. †Strain types identified through direct repeat unit typing.

.

Carriage of MRSP has not been identified in wild rats; therefore, the epidemiologic and public health implications of these findings are difficult to determine. However, the isolation of a common dog-associated *dru* type from rats suggests that MRSP might be transmissible between dogs and rats. This possibility is not surprising given the potential for direct and indirect contact between these species. Indeed, rat-to-dog transmission of other bacterial pathogens has been recognized (*9*). Detection of a *dru* type not previously detected in methicillin-resistant staphylococci suggests that these isolates might have evolved independently of methicillin-resistant staphylococci in other animal species.

Rat carriage of MRSP does not prove that rats are capable of transmitting the bacterium to humans; however, rats are a source of other zoonotic pathogens (*6*). These pathogens are most commonly transmitted from rats to humans indirectly, through contamination of the environment or foodstuffs (*6*). Of note, MRSP has been shown to survive for extended periods in the environment (*4*), suggesting a mechanism through which the bacterium could be passed from rats to humans and dogs or vice versa.

In 2011, MRSA was detected in bedbugs from Vancouver’s Downtown Eastside (*10*). We have identified another urban pest in this area as a potential source of a multidrug-resistant pathogen. As with bedbugs, rat infestations are most common in impoverished, inner-city neighborhoods, and residents of these neighborhoods are at greatest risk for rat-to-human pathogen transmission. These findings suggest that inner-city urban rats warrant further investigation as a potential source of MRSP and other contemporary zoonotic pathogens, including multidrug-resistant bacteria.
